# Supplementary material for: Hybrid Models and Biological Model Reduction with PyDSTool
Source: PLoS Comput Biol. 2012 Aug 9;8(8):e1002628. doi: 10.1371/journal.pcbi.1002628 (PMC3415397; doi:10.1371/journal.pcbi.1002628)
Supplement: Text S4 — Complete source code for the PyDSTool package (version 0.88.120504). Includes API documentation and help files linking to web pages. This file is identical to the current public release on Sourceforge.net. (ZIP) [file pcbi.1002628.s004.zip › PyDSTool/html/PyDSTool.Toolbox.optimizers.line_search.fixed_last_step_modifier-module.html]

xml version="1.0" encoding="ascii"?


PyDSTool.Toolbox.optimizers.line\_search.fixed\_last\_step\_modifier


| Home | Trees | Indices | Help | PyDSTool |
| --- | --- | --- | --- | --- |

Package PyDSTool ::
Package Toolbox ::
Package optimizers ::
Package line\_search ::
Module fixed\_last\_step\_modifier

[hide private]

[frames] | no frames]

# Module fixed\_last\_step\_modifier

source code

Line search decorator that overrides the default alpha\_step value with
a factor times the last alpha\_step


|  |  |
| --- | --- |
| Classes | [hide private] |

FixedLastStepModifier  
Overrides the default step size and replaces it with a factor times
the last one

| Home | Trees | Indices | Help | PyDSTool |
| --- | --- | --- | --- | --- |

|  |  |
| --- | --- |
| Generated by Epydoc 3.0.1 on Fri May 4 15:24:05 2012 | http://epydoc.sourceforge.net |
